# HSF2BP protects against acute liver injury by regulating HSF2/HSP70/MAPK signaling in mice

**DOI:** 10.1038/s41419-022-05282-x

**Published:** 2022-09-27

**Authors:** Jianbin Bi, Jia Zhang, Mengyun Ke, Tao Wang, Mengzhou Wang, Wuming Liu, Zhaoqing Du, Yifan Ren, Shuqun Zhang, Zheng Wu, Yi Lv, Rongqian Wu

**Affiliations:** 1 National Local Joint Engineering Research Center for Precision Surgery & Regenerative Medicine, Shaanxi Provincial Center for Regenerative Medicine and Surgical Engineering, The First Affiliated Hospital of Xi’an Jiaotong University, Xi’an, Shaanxi Province China; 2 Department of Oncology, The Second Affiliated Hospital of Xi’an Jiaotong University, Xi’an, Shaanxi Province China; 3 Department of Gastroenterology, The Second Affiliated Hospital of Xi’an Jiaotong University, Xi’an, Shaanxi Province China; 4 Department of Hepatobiliary Surgery, The First Affiliated Hospital of Xi’an Jiaotong University, Xi’an, Shaanxi Province China; 5 Department of Hepatobiliary Surgery, Shaanxi Provincial People’s Hospital, Xi’an, Shaanxi Province China; 6 Department of General Surgery, The Second Affiliated Hospital of Xi’an Jiaotong University, Xi’an, Shaanxi Province China

**Keywords:** Hepatotoxicity, Post-translational modifications

## Abstract

Heat shock proteins (HSPs) depletion and protein misfolding are important causes of hepatocyte death and liver regeneration disorder in liver injury. HSF2BP, as its name implies, is a binding protein of HSF2, but the specific role of HSF2BP in heat shock response (HSR) remains unknown. The aim of this study is to identify the role of HSF2BP in HSR and acute liver injury. In this study, we found that HSF2BP expression increased significantly within 24 h after APAP administration, and the trend was highly consistent with that of HSP70. *hsf2bp*-*KO* and *hsf2bp*-*TG* mouse models demonstrated HSF2BP reduced hepatocyte death, ameliorated inflammation, and improved liver function in APAP- or D-GalN/LPS- induced liver injury. Meanwhile, a significant increase of the survival rate was observed in *hsf2bp*-*TG* mice after APAP administration. Further studies showed that HSF2BP upregulated the expression of HSF2 and HSP70 and inhibited the activation of Jnk1/2 and P38 MAPK. Additionally, HSP70 siRNA pretreatment abolished the effect of HSF2BP on the MAPK pathway in APAP-treated hepatocytes. The results reveal that HSF2BP is a protective factor in acute liver injury, and the HSF2BP/HSP70/MAPK regulatory axis is crucial for the pathogenesis of liver injury. HSF2BP is a potential therapeutic target for liver injury.

## Introduction

Acute liver injury is a common clinical complication caused by a variety of factors, such as viral infection, improper drug use, excessive alcohol intake, and sepsis [1, 2]. Its severe form, acute liver failure, is a severe consequence of abrupt massive hepatocyte death, evolving over days or weeks to a lethal outcome, with a clinical mortality of 25–75% [2]. It is well known that the liver is an important organ for protein, lipid, and glucose metabolism and synthesizes most plasma proteins. Improper drug use, severe infection and other stimuli trigger the inflammatory cytokine storm, oxidative stress and mitochondrial dysfunction, resulting in protein misfolding and hepatocyte death [3–5].

Heat shock response (HSR) is an important protective mechanism of hepatocytes in harmful conditions [6]. Heat shock proteins (HSPs) are highly conserved stress proteins, promoting protein molecular folding and assembly, and refolding or removing misfolded proteins [7]. Plenty of studies have confirmed that overexpression of HSPs alleviates hepatocyte death and liver injury [8–12]. For example, overexpression of HSP72 in mouse livers alleviates multiple stress-induced liver injury, and sodium arsenite prevents ischemia-reperfusion injury by upregulating hepatocyte HSP70 expression [8, 10]. Additionally, HSPs play crucial roles in liver regeneration [13].

The MAPK family is important to signal transmitters from the cell surface to the nucleus, regulating cell proliferation and differentiation, cell stress, and apoptosis [14]. A large number of studies have confirmed that activation of MAPK is a key event in liver injury and liver regeneration [15–17]. Recent studies showed that HSPs regulated the activation of MAPK in liver diseases [10].

Heat shock factor 2 binding protein (HSF2BP) is initially isolated from the human testicular cDNA library, which can directly bind to HSF2 [18]. However, the role of HSF2BP in HSR is still unclear. Meanwhile, the role of HSF2BP in liver injury and repair remains unknown. Based on the above evidence, we hypothesized that HSF2BP alleviates liver injury by modulating the HSR. The aim of the present study is to clarify the role of HSF2BP in liver injury and its specific mechanisms.

## Results

### HSF2BP expression and HSR in liver injury

**Fig. 1:**
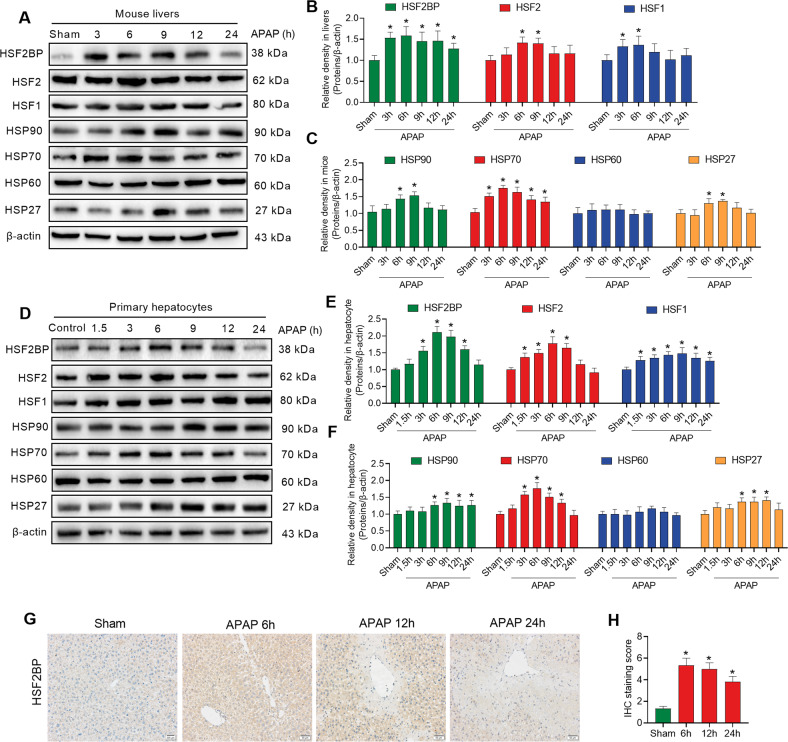
HSF2BP expression and heat shock response (HSR) in acetaminophen (APAP)-induced liver injury. Wild-type (WT) mice were intraperitoneally injected with 500 mg/kg APAP, and liver samples were harvested at 0, 3, 6, 9, 12, and 24 h after APAP administration. **A**–**C** Western blot analysis of the expression of HSF2BP, HSF2, HSF1, HSP90, HSP70, HSP60, and HSP27 in liver samples. *P < 0.05 versus the sham group; *n* = 6 per group. Primary hepatocytes were isolated and treated with 5 mM APAP. **D**–**F** Western blot analysis of the expression of HSF2BP, HSF2, HSF1, HSP90, HSP70, HSP60, and HSP27 at 0, 3, 6, 9, 12, and 24 h after APAP administration in hepatocytes. *P < 0.05 versus the sham group; *n* = 3 per group; **G**, **H** Immunohistochemistry analysis of liver HSF2BP expression. *P < 0.05 versus the sham group; *n* = 6 per group. mean ± SEM; The *t*-test was used to analyze the differences between the two groups.

To investigate the changes in HSF2BP expression and HSR in liver injury, the mice and primary hepatocytes were administrated with APAP. We found that HSF2BP expression in liver sample were increased after APAP administration in mice (Fig. 1A, B). Quantitative results of HSR-associated proteins suggested that HSF2, HSF1, HSP90, HSP70, and HSP27 in the liver sample exhibited varying degrees of elevation after APAP administration at certain times, while HSP60 expression did not altered (Fig. 1A–C). Similar results were seen in primary hepatocytes. Compared with the PBS-administration, APAP-treated hepatocytes increased HSF2BP expression at 3, 6, 9, and 12 h, but not 24 h (Fig. 1D–F). The expression trends of HSF2BP and HSP70 were highly consistent. Immunohistochemistry showed that HSF2BP increased in livers after APAP treatment, especially at 6 h after administration (Fig. 1G, H). Moreover, a prominent increase in HSF2BP expression was observed in the livers of patients with the benign end-stage liver disease compared with the normal livers (Fig. S1). These results suggest that HSF2BP expression is increased in liver injury and is closely related to HSR.

### Liver-specific *hsf2bp* gene deletion aggravates liver injury in mice

**Fig. 2:**
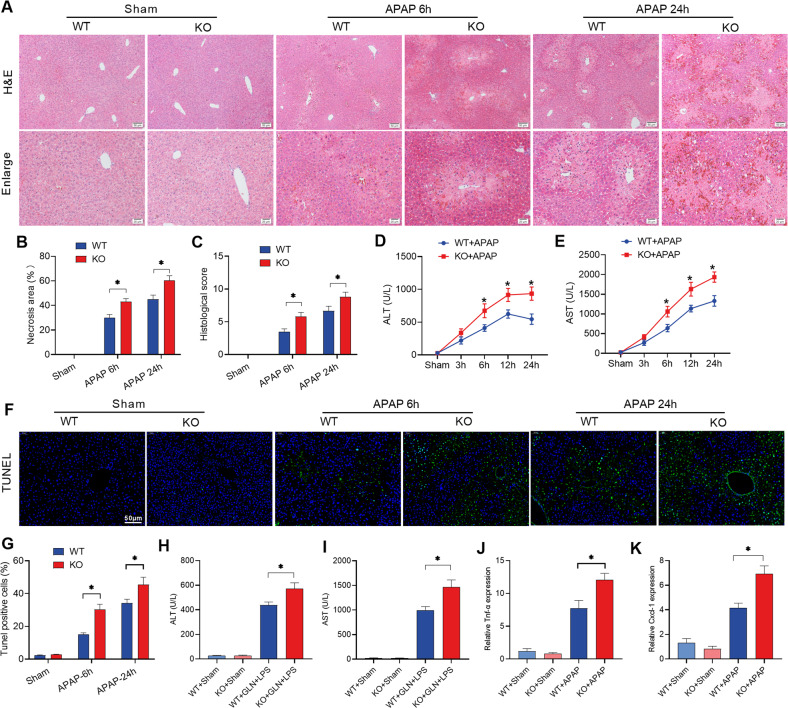
Liver-specific hsf2bp gene deletion aggravates liver injury in mice. WT mice and liver-specific *hsf2bp*^*−/−*^ mice were intraperitoneally injected with 500 mg/kg APAP, and liver samples and blood samples were harvested at 0, 3, 6, 12, and 24 h after APAP administration. **A** Hematoxylin and eosin staining (H&E) of representative liver sections; **B** Percentage of necrotic areas; **C** Liver histological scores; **D**, **E** Serum alanine aminotransferase (ALT) and aspartate aminotransferase (AST) activity; **F**, **G** TUNEL fluorescence staining (green), the corresponding nuclear counterstaining (blue) and percentage of TUNEL positive cells. WT and liver-specific *hsf2bp*^*−/−*^ mice were intraperitoneally injected with 700 mg/kg D-GalN + 10 μg/kg LPS, and blood samples were harvested at 0 h and 6 h. **H**, **I** Serum ALT and AST activity. **J**, **K** qPCR analysis of *Tnf-α* and *Cxcl-1* expression at 6 h after APAP administration. *n* = 6, mean ± SEM, *P < 0.05 versus WT group. The *t*-test was used to analyze the differences between the two groups.

To further explore the role of HSF2BP in liver injury, liver-specific *hsf2bp* gene knockout mice were administrated with 500 mg/kg APAP or 700 mg/kg D-GalN + 10 μg/kg LPS. The histological results showed large necrosis and inflammatory cell infiltration in livers after APAP treatment, and the liver-specific *hsf2bp*^*−/−*^ mice exhibited more severe liver injury than WT mice (Fig. 2A). Further quantitative analysis revealed that liver necrosis area and the histological score of *hsf2bp*^−*/−*^ mice were higher than those of WT mice after APAP administration (Fig. 2B, C). Meanwhile, *hsf2bp*^*−/*−^ mice showed higher serum ALT and AST levels than WT mice after APAP treatment at 3, 6, 12, and 24 h, respectively (Fig. 2D, E). Compared with the WT group, livers from the *hsf2bp*^*−/*−^ mice exerted stronger positive TUNEL staining, suggesting HSF2BP plays an anti-apoptosis role in APAP-induced liver injury (Fig. 2F, G). Similar results were shown in the D-GalN/LPS-induced liver injury model. We observed more severe liver dysfunction in *hsf2bp*^*−/−*^ mice than that in WT mice after D-GalN/LPS administration (Fig. 2H, I). Moreover, qPCR results suggested the liver *tfn-α* and *cxcl-1* expression were higher in *hsf2bp*^*−/*−^ mice than those in WT mice (Fig. 2J, K). These results confirmed that loss of HSF2BP aggravates liver injury.

### Liver-specific HSF2BP overexpression alleviates liver injury in mice

**Fig. 3:**
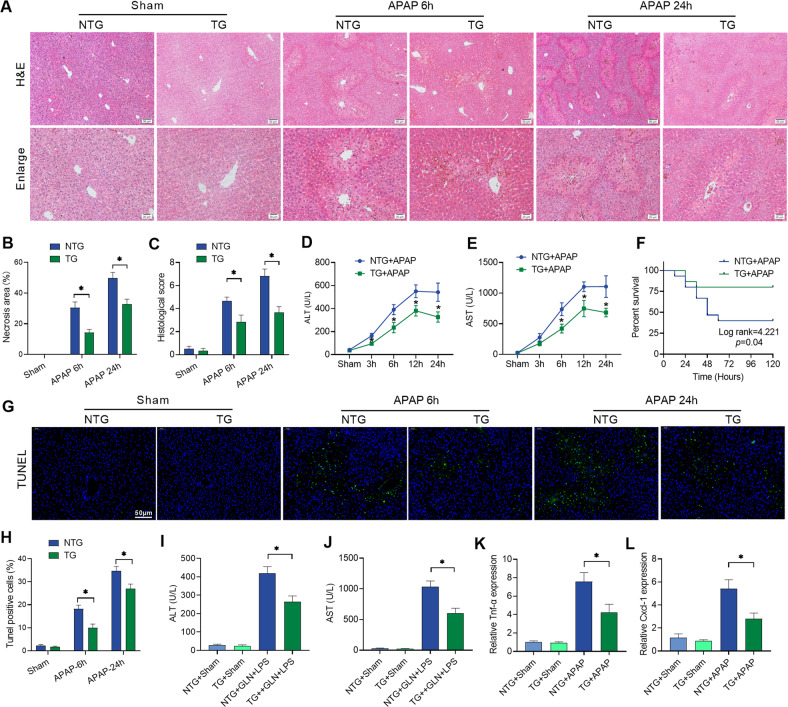
Liver-specific HSF2BP overexpression alleviates liver injury in mice. Liver-specific *hsf2bp-*TG mice and NTG mice were intraperitoneally injected with 500 mg/kg APAP, and liver samples and blood samples were harvested at 0, 3, 6, 12, and 24 h after APAP administration. **A** Hematoxylin and eosin staining (H&E) of representative liver sections; **B** Percentage of necrotic areas; **C** Liver histological scores; **D**, **E** Serum ALT and AST activity; **F** Liver-specific *hsf2bp-*TG mice and NTG mice were intraperitoneally injected with 750 mg/kg APAP. Survival analysis was conducted by Kaplan–Meier curves and log-rank testing. **G**, **H** TUNEL fluorescence staining (green), the corresponding nuclear counterstaining (blue) and percentage of TUNEL positive cells. WT mice and liver-specific *hsf2bp-*TG mice were intraperitoneally injected with 700 mg/kg D-GalN + 10 μg/kg LPS, and blood samples were harvested at 0 and 6 h. **I**, **J** Serum ALT and AST activity. **K**, **L** qPCR analysis of *Tnf-α* and *Cxcl-1* expression at 6 h after APAP administration. *n* = 6, mean ± SEM, *P < 0.05 versus NTG group. The *t*-test was used to analyze the differences between the two groups.

We used liver-specific *hsf2bp-TG* mice overexpressing *hsf2bp* in hepatocytes to further investigate the function of HSF2BP in injury. Liver necrotic area, inflammatory cell infiltration, histological score, serum ALT and AST levels were all decreased in *hsf2bp-TG* mice compared with NTG mice (Fig. 3A–E). Meanwhile, 750 mg/kg APAP were administrated in *hsf2bp-TG* and NTG mice for survival analysis. A prominent increase in survival rate was observed in *hsf2bp-TG* mice compared with NTG mice after APAP treatment (Fig. 3F). TUNEL staining showed HSF2BP overexpression significantly reduced hepatocyte apoptosis at 6 and 24 h in APAP-induced liver injury (Fig. 3G, H). Consistent with the above results, *hsf2bp-TG* mice exhibited lower serum ALT and AST activity at 6 h after D-GalN/LPS administration (Fig. 3I, J). Additionally, qPCR results suggested the liver *tfn-α* and *cxcl-1* expression were lower in *hsf2bp-TG* mice than those in NTG mice (Fig. 3K, L). Our results suggested that *hsf2bp* overexpression alleviates liver injury.

### HSF2BP upregulates HSF2/HSP70 signaling in APAP-treated mice

**Fig. 4:**
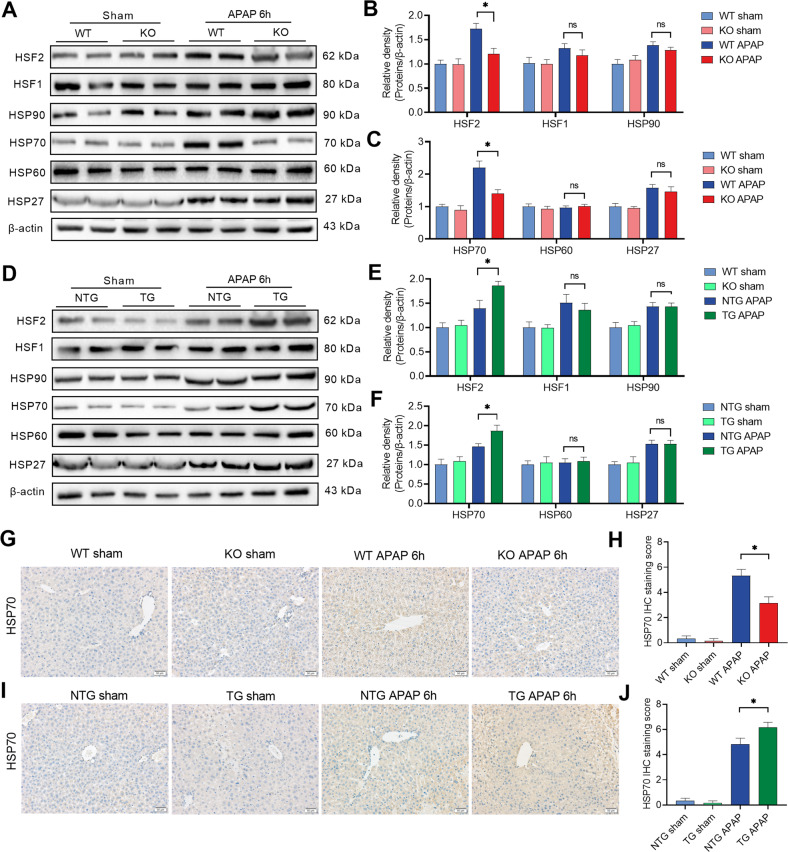
HSF2BP upregulates HSF2/HSP70 signaling in mice. WT mice and liver-specific *hsf2bp*^*−/−*^ mice were intraperitoneally injected with 500 mg/kg APAP, and liver samples were harvested at 0 and 6 h after APAP administration. **A**–**C** Western blot analysis of the expression of HSF2, HSF1, HSP90, HSP70, HSP60, and HSP27 in liver samples; *P < 0.05 versus the WT + APAP group. NTG mice and liver-specific *hsf2bp-*TG mice were intraperitoneally injected with 500 mg/kg APAP, and liver samples were harvested at 0 and 6 h after APAP administration. **D**–**F** Western blot analysis of the expression of HSF2, HSF1, HSP90, HSP70, HSP60, and HSP27 in liver samples. *P < 0.05 versus the NTG + APAP group. **G**, **H** Immunohistochemistry analysis of the liver HSF2BP expression in WT mice and liver-specific *hsf2bp*^*−/*−^ mice; *P < 0.05 versus the WT + APAP group; **I**, **J** Immunohistochemistry analysis of the liver HSF2BP expression in NTG mice and liver-specific *hsf2bp-*TG mice; *P < 0.05 versus the NTG + APAP group; ns. means no difference, *n* = 6, mean ± SEM. The *t*-test was used to analyze the differences between the two groups.

Liver-specific *hsf2bp*^−/−^ mice and *hsf2bp-TG* mice were used to study the role of HSF2BP in HSR. We found that the expression of HSF2, HSF1, HSP90, HSP70, and HSP27 except HSP60, increased at 6 h after APAP treatment in liver samples. Meanwhile, HSF2 and HSP70 expression showed remarkable reductions in liver-specific *hsf2bp*^−/−^ mice compared with the WT mice after APAP administration (Fig. 4A–C). Meanwhile, the expression of HSF1, HSP90, HSP60, and HSP27 showed no difference between WT mice and *hsf2bp*^−/−^ mice. By contrast, *hsf2bp-TG* mice exhibited opposite results. A prominent increase in HSF2 and HSP70 expression was observed in liver-specific *hsf2bp-TG* mice compared with the NTG mice after APAP administration (Fig. 4D–F). Consistent with the western blot analysis, immunohistochemistry indicated that HSP70 expression was increased after APAP administration. Liver-specific *hsf2bp* gene deletion reduced liver HSP70 expression, while liver-specific HSF2BP overexpression increased liver HSP70 level at 6 h after APAP administration (Fig. 4G–J).

### HSF2BP restrains activation of Jnk and P38 MAPK in APAP-induced liver injury

**Fig. 5:**
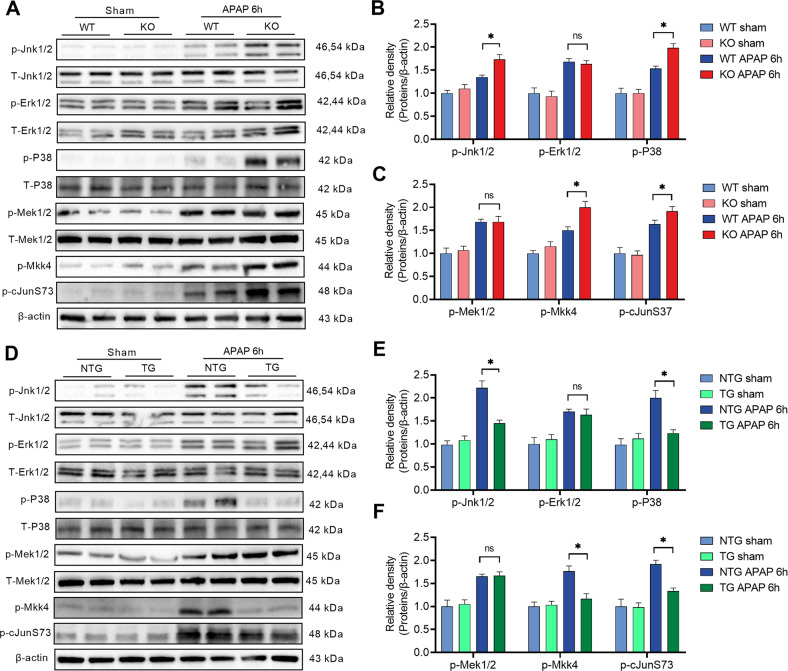
HSF2BP restrains activation of Jnk and P38 MAPK in APAP-induced liver injury. WT mice and liver-specific *hsf2bp*^*−/*−^ mice were intraperitoneally injected with 500 mg/kg APAP, and liver samples were harvested at 0 and 6 h after APAP administration. **A**–**C** Western blot analysis of the expression of Jnk1/2, p-Jnk1/2, Erk1/2, p-Erk1/2, P38, p-P38, T-Mek1/2, p-Mek1/2, p-MKK4, and p-cJunS73 in liver samples; *P < 0.05 versus the WT + APAP group. WT mice and liver-specific *hsf2bp-*TG mice were intraperitoneally injected with 500 mg/kg APAP, and liver samples were harvested at 0 and 6 h after APAP administration. **D**–**F** Western blot analysis of the expression of Jnk1/2, p-Jnk1/2, Erk1/2, p-Erk1/2, P38, p-P38, T-Mek1/2, p-Mek1/2, p-MKK4, and p-cJunS73 in liver samples. *P < 0.05 versus the NTG + APAP group. ns. means no difference, *n* = 6, mean ± SEM. The *t*-test was used to analyze the differences between the two groups.

MAPK pathway mediating the inflammatory response and cell death plays a crucial role in liver injury. We hypothesized that HSF2BP plays a protective role in liver injury by inhibition of MAPK phosphorylation. As reported in previous studies, APAP activated MAPK signaling, which is embodied in increased phosphorylation of Jnk1/2 (p-Jnk1/2), Erk1/2 (p-Erk1/2), P38 (p-P38), and Mek1/2 (p-Mek1/2) in mice. Meanwhile, MAPK activation-dependent kinase Mkk4 (p-Mkk4) and cJunS73 also increased after APAP administration (Fig. 5A–C). In contrast to the WT mice, liver-specific *hsf2bp*^−/−^ mice increased p-Jnk1/2, p-P38, upstream kinase Mkk4 (p-Mkk4) and cJunS73, but not p-Erk1/2 and p-Mek1/2, in APAP-induced liver injury (Fig. 5A–C). However, *hsf2bp-TG* mice exhibited the opposite situation. Liver-specific *hsf2bp* overexpression abolished the increase of p-Jnk1/2, p-P38, upstream kinase Mkk4 (p-Mkk4) and cJunS73 in APAP-treated mice (Fig. 5D–F). Taken together, these results indicated that HSF2BP protects against liver injury via inhibition of phosphorylation of Jnk1/2 and P38.

### HSF2BP regulates HSP70/MAPK pathway via binding to HSF2BP

**Fig. 6:**
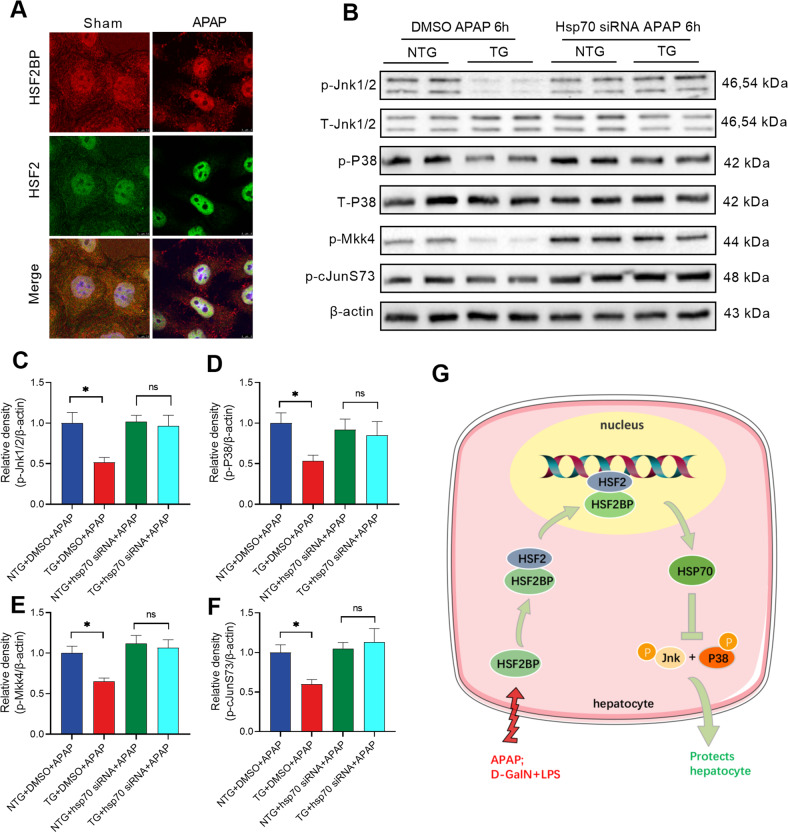
HSF2BP regulates HSP70/MAPK pathway by binding to HSF2. Primary hepatocytes were isolated and treated with 5 mM APAP. **A** immunofluorescent staining of HSF2 and HSF2BP at 6 h after APAP administration. Primary hepatocytes were pretreated with HSP70 siRNA. **B**–**F** Western blot analysis of the expression of Jnk1/2, p-Jnk1/2, P38, p-P38, p-MKK4, and p-cJunS73 in 5 mM APAP-treated hepatocytes. ns. means no difference, *n* = 6, mean ± SEM. The *t*-test was used to analyze the differences between the two groups. **G** HSF2BP protects against liver injury by regulating HSF2/HSP70/MAPK signaling.

The foregoing results confirmed that HSF2BP was related to HSR, and we further determined the hepatocellular localization of HSF2BP and HSF2 by immunofluorescence. We found that under normal conditions, HSF2BP and HSF2 were distributed in the nucleus and cytoplasm (Fig. 6A). At 2 h after 5 mM APAP treatment, HSF2BP and HSF2 almost all entered the nucleus (Fig. 6A). Consistent with previous literature reports, the distribution of HSF2BP is highly consistent with that of HSF2, suggesting that HSF2BP can directly bind to HSF2 in APAP-induced hepatocyte injury and further regulate transcription of downstream HSPs. To further confirm whether HSF2BP inhibits MAPK phosphorylation via regulating HSP70 expression, we used small interfering RNA to interfere with HSP70 expression in hepatocytes. The results indicated that in HSP70 siRNA pretreated hepatocytes, the expression of p-Jnk1/2, p-P38, p-Mkk4 and cJunS73 showed no difference between the *hsf2bp-TG* hepatocyte and NTG hepatocyte after APAP administration (Fig. 6B–F). The deletion of HSP70 abolished the effect of HSF2BP on the MAPK pathway, indicating HSF2BP restrains activation of Jnk and P38 MAPK via upregulating HSP70 expression.

## Discussion

HSPs depletion and protein misfolding are important causes of hepatocyte death and liver regeneration disorder in liver injury [10, 13]. In this study, we found HSF2BP expression increased significantly within 24 h after APAP administration. Hepatocyte-specific *hsf2bp*-*KO* mice showed more severe hepatocyte death and inflammation and aggravated liver dysfunction in APAP and D-GalN/LPS-induced liver injury. Meanwhile, hepatocyte-specific *hsf2bp-TG* mice exhibited an opposite phenotype and had a higher survival rate. Further studies showed HSF2BP inhibited the activation of Jnk1/2 and P38 MAPK via upregulating the expression of HSP70 expression. HSF2BP/HSP70/MAPK regulatory axis is a potential therapeutic target for liver injury.

HSF2BP is a protein that can directly bind to HSF2, initially isolated from a human testis cDNA library by a yeast two-hybrid system [18]. Since its discovery in 1998, the role of HSF2BP is still poorly understood. The previous studies found that HSF2BP can also bind to BRCA2 and BNC1, playing different roles in cellular activity [19–21]. Current reports of HSF2BP mainly focus on meiosis, spermatogenesis and homologous recombination repair [22, 23]. Besides, HSF2BP has been reported to be transcribed in all cultured human cancer cell lines and elevated in some tumor samples [19, 24]. Inactivation of the *hsf2bp* gene in mice leads to male infertility [25]. This study found that the expression of HSF2BP was increased in livers and hepatocytes after APAP treatment both in vivo and in vitro study. In particular, the increase of HSF2BP mainly appeared at 6 and 12 h and then gradually decreased. At 24 h, there showed no difference compared with the control group in APAP-treated hepatocytes. Therefore, we guessed that the increase of HSF2BP within 24 h was compensatory, and it was gradual exhausted as APAP stimulation continues. More importantly, the increased expression of HSF2BP is highly consistent with that of HSP70, suggesting that HSF2BP may be involved in the HSR and have a close relationship with HSP70.

HSR is a defensive adaptive response characterized by changes in HSP gene expression, which promotes the folding and assembly of protein molecules, as well as the refolding or removal of misfolded proteins [7]. In recent years, abundant evidence proved that HSPs are closely related to liver injury [8, 10, 12, 26]. HSP70 family is the main intracellular HSP system, which plays a key role in protein transport and folding [27]. A recent study reported that HSP72, a member of the HSP70 family, is markedly increased in liver patients, and overexpression of HSP72 in mouse liver reduces multiple cause-induced liver injury [10]. Besides, sodium arsenite prevents ischemia-reperfusion injury by upregulating HSP70 expression in mouse liver [8]. HSF2BP, as its name implies, is a binding protein of HSF2, but the specific role of HSF2BP in HSR has not been reported. In this study, we constructed *hsf2bp*-*KO* and *hsf2bp*-*TG* mice and proved that HSF2BP reduced hepatocytes death, ameliorated inflammation, and improved liver function in APAP and D-GalN/LPS-induced liver injury. Meanwhile, we found HSF2BP upregulated the expression of HSF2 and HSP70, and HSP70 siRNA pretreatment abolished the effect of HSF2BP on the MAPK pathway in APAP-treated hepatocytes. The results indicated that HSF2BP protects against liver injury by regulating the HSP70-related HSR.

HSF1 and HSF2 are considered the most important heat shock factors (HSFs) [28]. Under normal conditions, HSF maintains a monomer structure in the cytoplasm. In response to heat shock or other forms of stress, HSF is assembled into trimers that enter the nucleus and binds to HSE located in the promoter region to promote the transcription of *hsp* mRNA [29]. Previous studies showed that HSF2 promotes the transcription of HSP70, but in the absence of HSF1, the transcription promoting effect of HSF2 on HSP70 disappears, suggesting that HSF2 and HSF1 may have a synergistic effect in the activation of HSP70 transcription [30–33]. In addition, the expression of HSF2 in the liver and heart is significantly higher than that in other tissues, suggesting HSF2 may play a non-negligible role in the stress response of the liver and heart [34]. In this study, we found that HSF2BP upregulated the expression of liver HSF2 and HSP70 in APAP-treated mice. Meanwhile, the distribution of HSF2BP is highly consistent with that of HSF2 in APAP-induced hepatocyte injury, suggesting that HSF2BP upregulated HSP70 expression via binding to the HSF2. However, whether HSF2BP regulates the HSP70 expression by participating in the interaction between HSF1 and HSF2 remains unclear and further studies are needed in the future.

MAPK family, including Erk1/2, Jnk1/2, and P38, are important signal transmitters from the cell surface to the nucleus, regulating cell proliferation and differentiation, cell stress, apoptosis, and other pathophysiological processes [14]. A large number of studies have confirmed that MAPK plays a crucial role in liver diseases [10, 16, 35]. For example, Jnk activation directly causes hepatocyte death during APAP-induced liver injury, and compound leflunomide attenuates APAP-induced liver injury by inhibition of Jnk phosphorylation [3, 36]. P38 MAPK is strongly activated by a variety of stress and plays important roles in inflammation and regulation of cell survival and differentiation [37]. In addition, an abundance of evidence proved that the MAPK pathway is regulated by HSPs [38, 39]. For example, HSP72 reduces cell death and oxidative stress in liver injury by inhibition of Jnk phosphorylation. In this study, we found that HSF2BP upregulated the expression of HSF2 and HSP70 and inhibited the activation of Jnk1/2 and P38 MAPK. HSP70 siRNA pretreatment abolished the effect of HSF2BP on the MAPK pathway in APAP-treated hepatocytes, suggesting HSF2BP protects against liver injury by regulating HSP70/MAPK signaling.

There are some limitations that should be mentioned in this study. Firstly, the present study is only based on animal experiments, and further clinical trials are needed. Secondly. we found that HSF2BP promoted the transcription of HSP70 via binding to HSF2. However, whether HSF2BP participates in the interaction between HSF1 and HSF2 needs further study. Finally, previous studies proved that HSF2BP can bind to HSF2, BRCA2, and BNC1 [19–21]. This study mainly focused on the role of HSF2BP in HSF2-related HSR, and other pathways need further study.

In conclusion, HSF2BP/HSP70/MAPK regulatory axis is crucial for the pathogenesis of acute liver injury. Targeting HSF2BP might be a novel strategy for the prevention or treatment of the acute liver injury.

## Materials and methods

Detailed methods and full-length western blots are provided in the supplementary materials.

### Experimental animals

In this study, all the animal experiments were performed on male wild-type C57BL/6 J mice, liver-specific *hsf2bp-TG* mice and liver-specific *hsf2bp-KO* mice (aged 6–8 weeks, weighing 20–25 g). Liver-specific *hsf2bp-TG* mice *w*ere constructed by inserting the ALB-Mouse Hsf2bp CDS-Polya fragment at Rosa26 on chromosome 6 with the CRISPR-Pro gene knockin technique. The *hsf2bp-TG* mice were identified by PCR with primer sequence: forward: 5′-GTTCACGGTCCATTATCAGTTG-3′, reverse: 5′-CACTGAAATGCTCAAATGGGAG-3′; Liver-specific *hsf2bp-KO* mice were constructed by CRISPR-Pro gene knockout technique and identified by PCR with primer sequence: forward: 5′-ACCATGTGGCAGTGAATATCCCAGA-3′, reverse: 5′-CCAGGGAAGGGAAGGAGTCTTCTA-3′. All experimental operations were performed in accordance with the requirements of the China Council on Animal Care and Use, and all experimental protocols were approved by the Ethics Committee of Xi’an Jiaotong University Health Science Center. In all experiments, mice were euthanized after anesthesia with isoflurane to minimize pain in this study.

### Mouse models of liver injury

The liver failure model was established in mice by intraperitoneal injection of 500 mg/kg APAP (A105809, Aladdin, Shanghai, China) or 700 mg/kg D-GalN (G115553, Aladdin, Shanghai, China) + 10 μg/kg LPS (055:B5, L118716 Aladdin, Shanghai, China). In survival analysis, 750 mg/kg APAP were administrated in *hsf2bp-TG* and *NTG* mice for survival analysis.

### Statistical analysis

All measurement data were expressed as the means ± standard error (SEM). The difference between groups was analyzed by *t*-test or one-way ANOVA. Kaplan–Meier curves and log-rank testing were used for survival analysis. All statistical analyses were performed with SPSS 18.0 software. *p* < 0.05 represented a significant difference.

## Supplementary information

Supplementary material

Full uncut gels

checklist

## Data Availability

Data were available on request.
