# Supplementary material for: HSF2BP protects against acute liver injury by regulating HSF2/HSP70/MAPK signaling in mice
Source: Cell Death Dis. 2022 Sep 27;13(9):830. doi: 10.1038/s41419-022-05282-x (PMC9515097; doi:10.1038/s41419-022-05282-x)
Supplement: Supplementary file 1 — Supplementary material [file 41419_2022_5282_MOESM1_ESM.docx]

***Supplemental Material for***

**HSF2BP protects against acute liver injury by regulating HSF2/HSP70/MAPK Signaling in mice**

Jianbin Bi, Jia Zhang, Mengyun Ke, Tao Wang, Mengzhou Wang, Wuming Liu, Zhaoqing Du, Yifan Ren, Shuqun Zhang, Zheng Wu, Yi Lv, Rongqian Wu*

Supplementary Figures


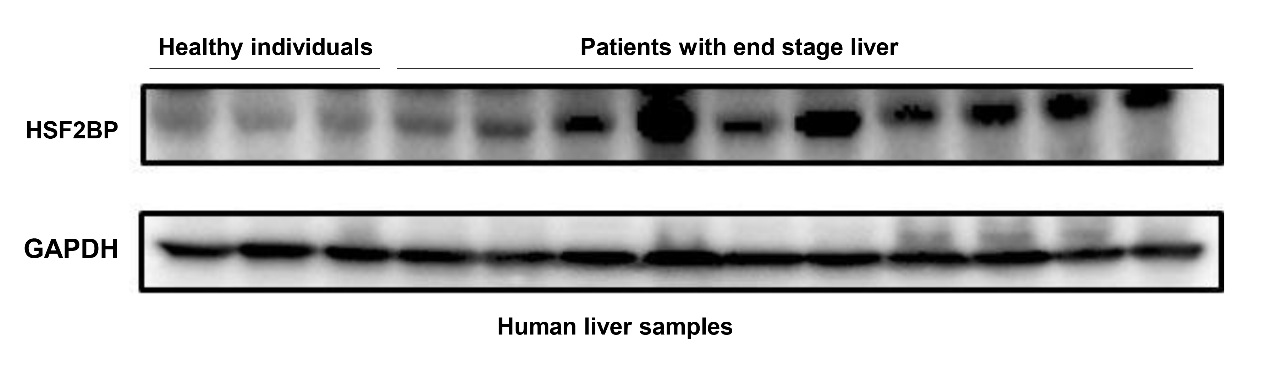


**Figure S1. HSF2BP expression in patients with benign end-stage liver diseases.** Human liver samples of healthy controls and patients with end stage liver diseases were obtained from the First Affiliated Hospital of Xi’an Jiaotong University, China. Liver tissues from hepatic surgical patients without hepatitis, steatosis, and I/R injury were used as healthy controls. Samples of benign end-stage liver disease were obtained from diseased liver of liver transplant patients. All subjects signed the informed consent forms and approved by Internal Review Board of the First Affiliated Hospital of Xi’an Jiaotong University. Patients were not involved in the design, conduct, reporting or dissemination plans of the study.

**Detailed methods**

**Primary hepatocyte extraction and culture**

Primary hepatocytes were isolated as described previously ^1^. Hepatocytes were cultured with DMEM/F12 medium, containing FBS, dexamethasone, insulin and penicillin-streptomycin solution. Hepatocyte viability was determined by trypan blue exclusion (cell viability >85% is available).

**Depletion of HSP70**

Hsp70-specific small interfering RNA (siRNA) was synthesized in GenePharma (Shanghai, China) with the following sequences: 5'-GGUCCUAAGAAUCGUUCAATT-3' and 5'-UUGAACGAUUCUUAGGACCTT-3'. Transfection with HSP70-siRNA for 48 h, and then hepatocytes were used for subsequent experiments.

**Western blot analysis**

Western blot analysis was performed as described previously ^2^. The PVDF membranes were incubated with the following primary monoclonal antibodies: rabbit anti-HSF2BP (ab126252,1:1000); rabbit anti-HSF2 (10706-1-AP, proteintech group,1:1000); rabbit anti-HSF1 (ab61382,abcam,1:1000); rabbit anti-HSP90 (ab203126,abcam,1:1000); rabbit anti-HSP70 (ab181606,abcam,1:1000); rabbit anti-HSP60 (ab190828,abcam,1:1000); rabbit anti-HSP27 (ab109376,abcam,1:1000); MAPK Family Antibody Sampler Kit(9926, Cell Signaling Technology,1:1000); Phospho-MAPK Family Antibody Sampler Kit (9910, Cell Signaling Technology,1:1000); rabbit anti-MEK1/2 (8727, Cell Signaling Technology,1:1000); rabbit anti-Phospho-MEK1/2 (Ser217/221) (3958, Cell Signaling Technology,1:1000); rabbit anti-Phospho-MKK4 (Ser257) (4514, Cell Signaling Technology,1:1000); rabbit anti-Phospho-c-Jun (Ser73) (3270, Cell Signaling Technology,1:1000); rabbit anti-β-Actin (4970, Cell Signaling Technology,1:1000) at 4°C overnight, and then incubated with HRP-conjugated Affinipure Goat Anti-Rabbit IgG (SA00001-2, Proteintech group, 1:5000). Proteins were quantified by ImageJ2x software and expressed as the relative intensity of protein/β-actin.

**Immunohistochemistry**

Immunohistochemistry was performed as described previously ^2^. Mouse livers were fixed with 4% paraformaldehyde and tissue slices were incubated with primary rabbit anti-HSF2BP (ab126252, abcam,1:500) and rabbit anti-HSP70 (ab181606,abcam,1:100). The staining score was calculated by the sum of staining intensity (0, negative; 1, mild; 2, moderate; and 3, severe), and percentage of positive cells (0, negative; 1, 1–25%; 2, 26–50%; 3, 51–75%; and 4, 76–100%), ranging from 0-7sd.

**Immunofluorescent**

Immunofluorescent was performed as described previously ^3^. Primary hepatocytes were immobilized with 4% paraformaldehyde and incubated with primary mouse HSF2BP (sc-130322, Santa, USA,1:20 dilution) and rabbit HSF2(sc-130322, proteintech group,1:200) overnight at 4°C. Then samples were incubated with Alexa Fluor 594–conjugated Goat Anti-Mouse IgG (SA00013-3, Proteintech, 1:200 dilution) and CoraLite488–conjugated Donkey Anti-Rabbit IgG (SA00013-6, Proteintech, 1:200 dilution) were incubated for 1 h at room temperature. The results were observed with a confocal microscope (TCS SP8 STED 3X, Leica, Germany).

**Histological analysis**

Liver tissues were fixed in 4% paraformaldehyde and Hematoxylin and eosin (HE) staining were performed as previously ^2^. Liver histological score was calculated as the sum of the individual score (0, no; 1, mild; 2, moderate; and 3 severe) of microvascular stasis of erythrocytes, nuclear fragmentation, nuclear fading, nuclear condensation, cytoplasmic color fading and cytoplasmic vacuolization. The total score ranges from 0-18.

**TUNEL**

Mouse liver paraffin sections were stained with transferase-mediated deoxyuridine triphosphate-biotin nick end labeling (TUNEL) kit (11684795910, Roche, Switzerland) according to the kit instructions.

**qPCR**

qPCR was performed as previously described ^3^. The liver total RNA was isolated by Trizol, and *tnf-α*, and *cxcl-1* mRNA expression were normalized to the *β-actin* mRNA. The primers were synthesized in Takara Biomedical Technology and sequences were as follows: mouse *tfn-α*: forward: 5'-GCCAGAGCCACATGCTCCTA-3', reverse: 5'-GATAAGGCTTGGCAACCCAAGTAA-3'; mouse *cxcl-1*: forward: 5'-TGCACCCAAACCGAAGTC-3', reverse: 5'-GTCAGAAGCCAGCGTTCACC-3'. The relative *tnf-α*, and *cxcl-1* mRNA expression were calculated with the Comparative-Ct Method (ΔΔCt method).

**Measurement of serum ALT and AST**

ALT activity analysis Kit (C009-2, NanJing JianCheng Bioengineering Institute, China) and AST activity analysis Kit (C010-2, NanJing JianCheng Bioengineering Institute China) were used for detection of serum ALT and AST activity according to the kit instructions.

**Reference**

1. Dong J, Ke MY, Wu XN, Ding HF, Zhang LN, Ma F*, et al.* SRY is a Key Mediator of Sexual Dimorphism in Hepatic Ischemia/Reperfusion Injury. *Ann Surg* 2020.

2. Bi J, Zhang J, Ren Y, Du Z, Li Q, Wang Y*, et al.* Irisin alleviates liver ischemia-reperfusion injury by inhibiting excessive mitochondrial fission, promoting mitochondrial biogenesis and decreasing oxidative stress. *Redox Biol* 2019, **20:** 296-306.

3. Bi J, Zhang J, Ren Y, Du Z, Zhang Y, Liu C*, et al.* Exercise hormone irisin mitigates endothelial barrier dysfunction and microvascular leakage-related diseases. *JCI Insight* 2020, **5**(13).
