# Supplementary material for: HSF2BP protects against acute liver injury by regulating HSF2/HSP70/MAPK signaling in mice
Source: Cell Death Dis. 2022 Sep 27;13(9):830. doi: 10.1038/s41419-022-05282-x (PMC9515097; doi:10.1038/s41419-022-05282-x)
Supplement: Supplementary file 2 — Full uncut gels [file 41419_2022_5282_MOESM2_ESM.pdf]

Figure 1A

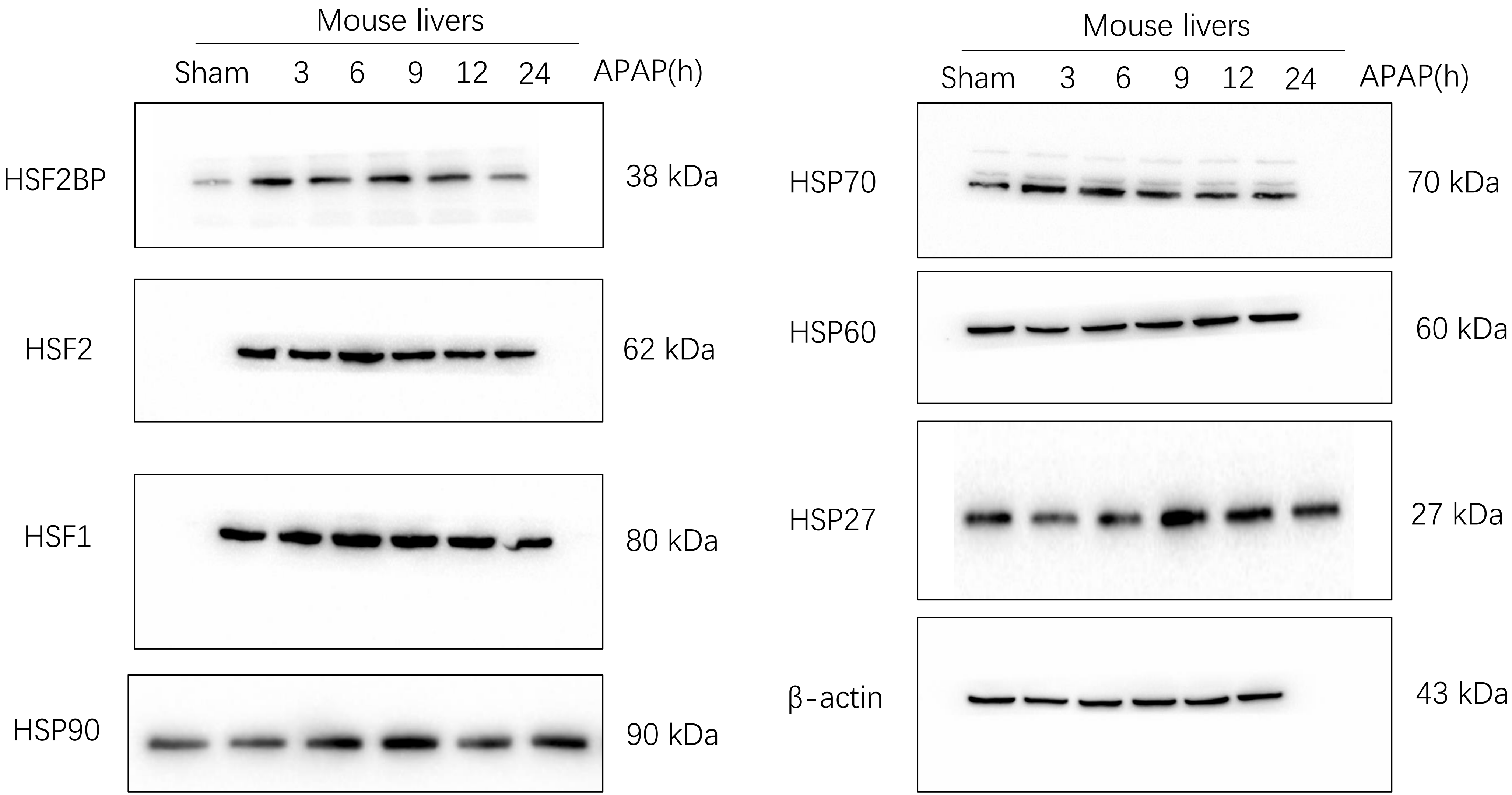
Mouse livers
Sham 3 6 9 12 24 APAP(h)
HSF2BP
38 kDa
HSF2
62 kDa
HSF1
80 kDa
HSP90
90 kDa
Mouse livers
Sham 3 6 9 12 24 APAP(h)
HSP70
70 kDa
HSP60
60 kDa
HSP27
27 kDa
β-actin
43 kDa

Figure 1D

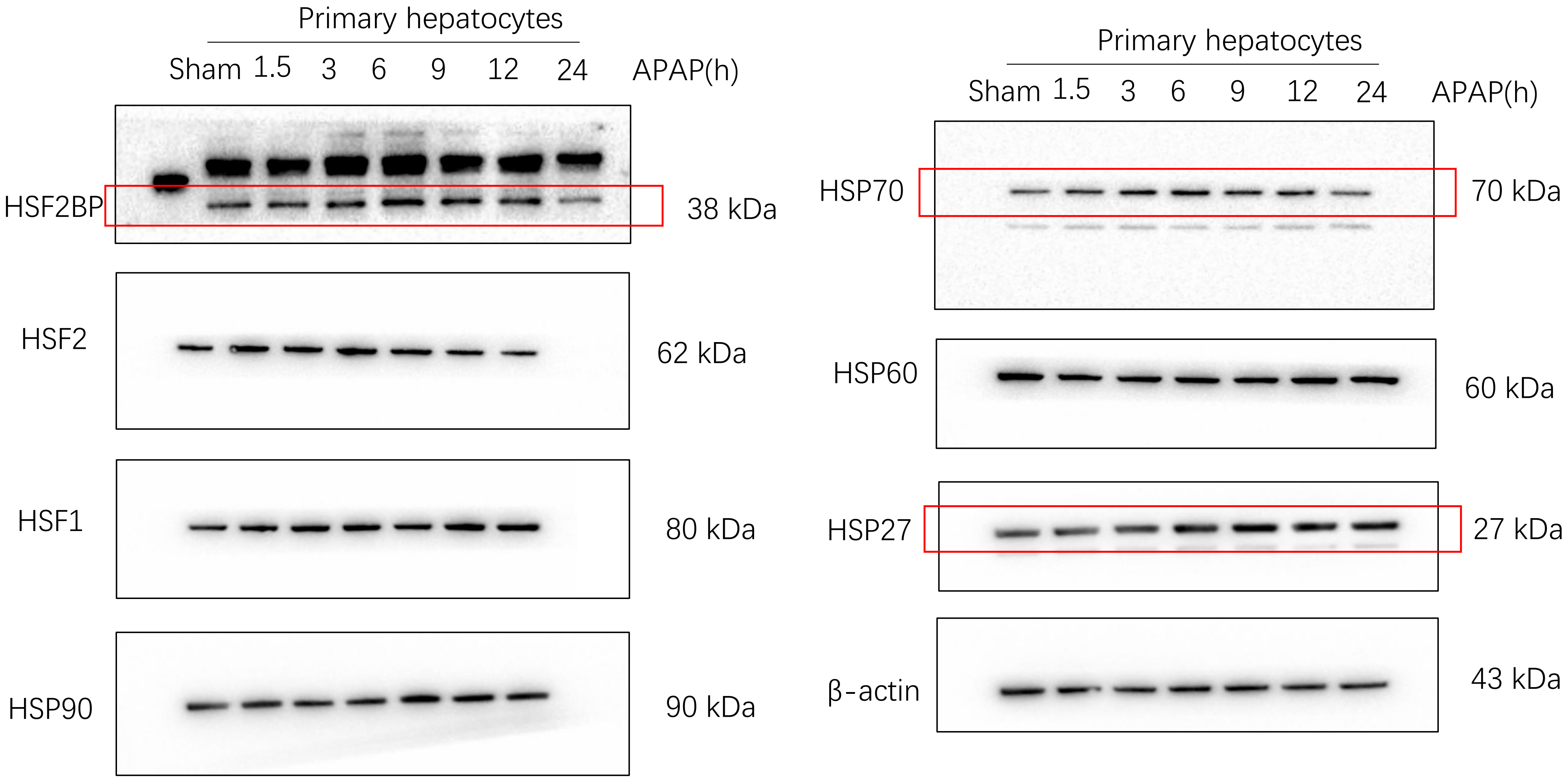

Primary hepatocytes
Sham 1.5 3 6 9 12 24 APAP(h)
HSF2BP
38 kDa
HSF2
62 kDa
HSF1
80 kDa
HSP90
90 kDa
Primary hepatocytes
Sham 1.5 3 6 9 12 24 APAP(h)
HSP70
70 kDa
HSP60
60 kDa
HSP27
27 kDa
β-actin
43 kDa

Figure 4A

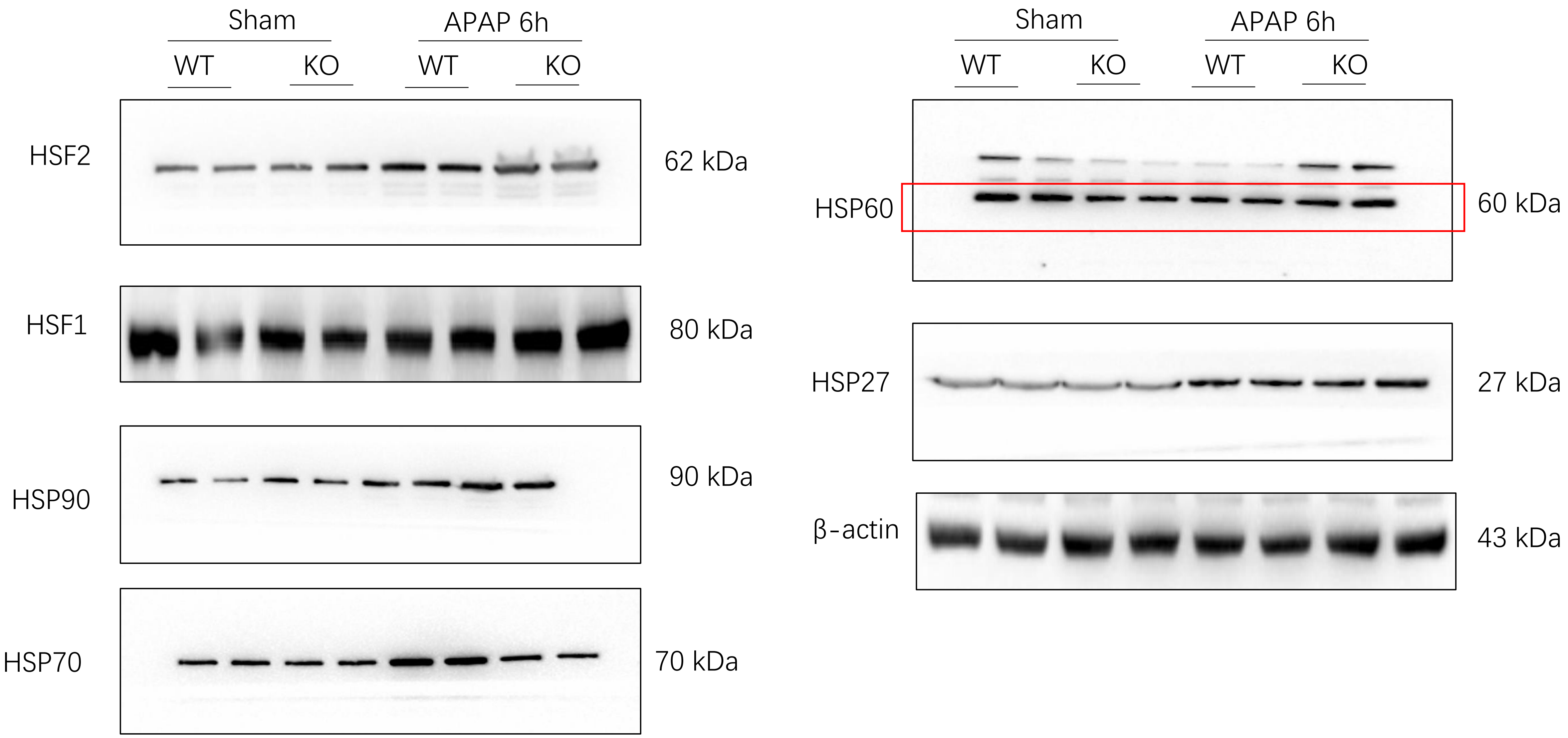
Sham
APAP 6h
WT
KO
WT
KO
HSF2
62 kDa
HSF1
80 kDa
HSP90
90 kDa
HSP70
70 kDa
Sham
APAP 6h
WT
KO
WT
KO
HSP60
60 kDa
HSP27
27 kDa
β-actin
43 kDa

Figure 4D

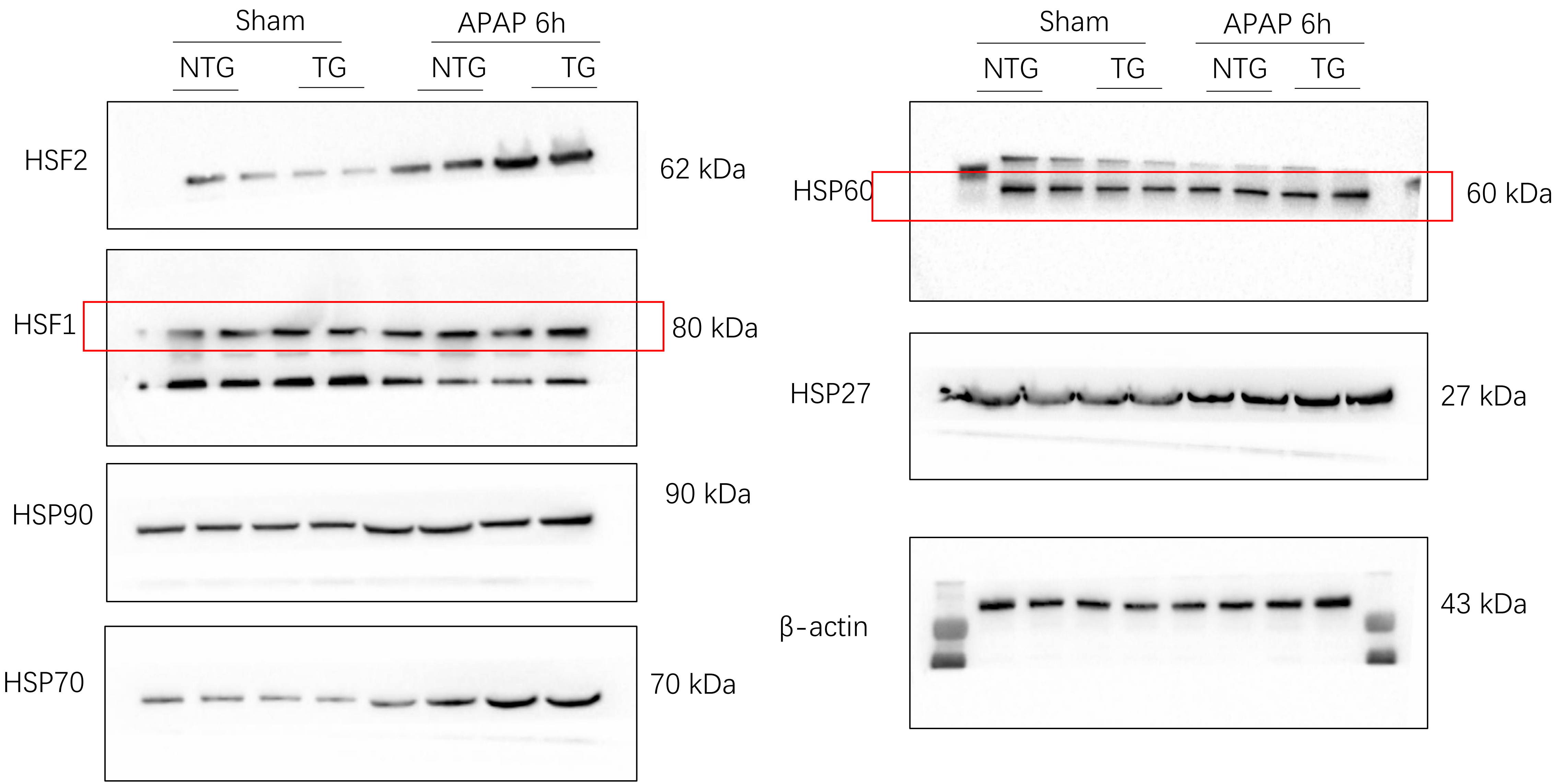

Sham
APAP 6h
NTG
TG
NTG
TG
HSF2
62 kDa
HSF1
80 kDa
HSP90
90 kDa
HSP70
70 kDa
Sham
APAP 6h
NTG
TG
NTG
TG
HSP60
60 kDa
HSP27
27 kDa
β-actin
43 kDa

Figure 5A

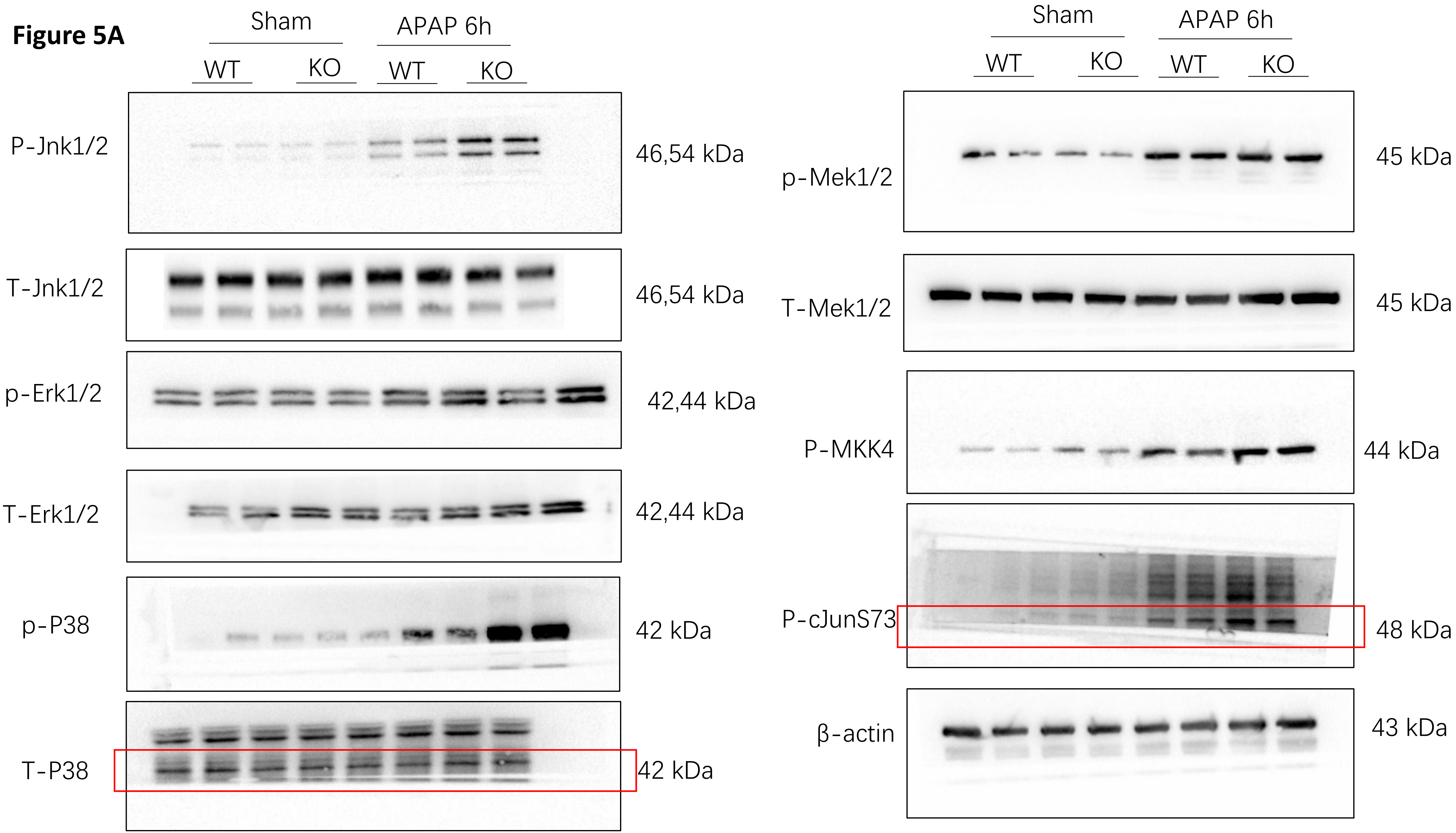

Sham
APAP 6h
WT
KO
WT
KO
P-Jnk1/2
46,54 kDa
T-Jnk1/2
46,54 kDa
p-Erk1/2
42,44 kDa
T-Erk1/2
42,44 kDa
p-P38
42 kDa
T-P38
42 kDa
Sham
APAP 6h
WT
KO
WT
KO
p-Mek1/2
45 kDa
T-Mek1/2
45 kDa
P-MKK4
44 kDa
P-cJunS73
48 kDa
β-actin
43 kDa

Figure 5D

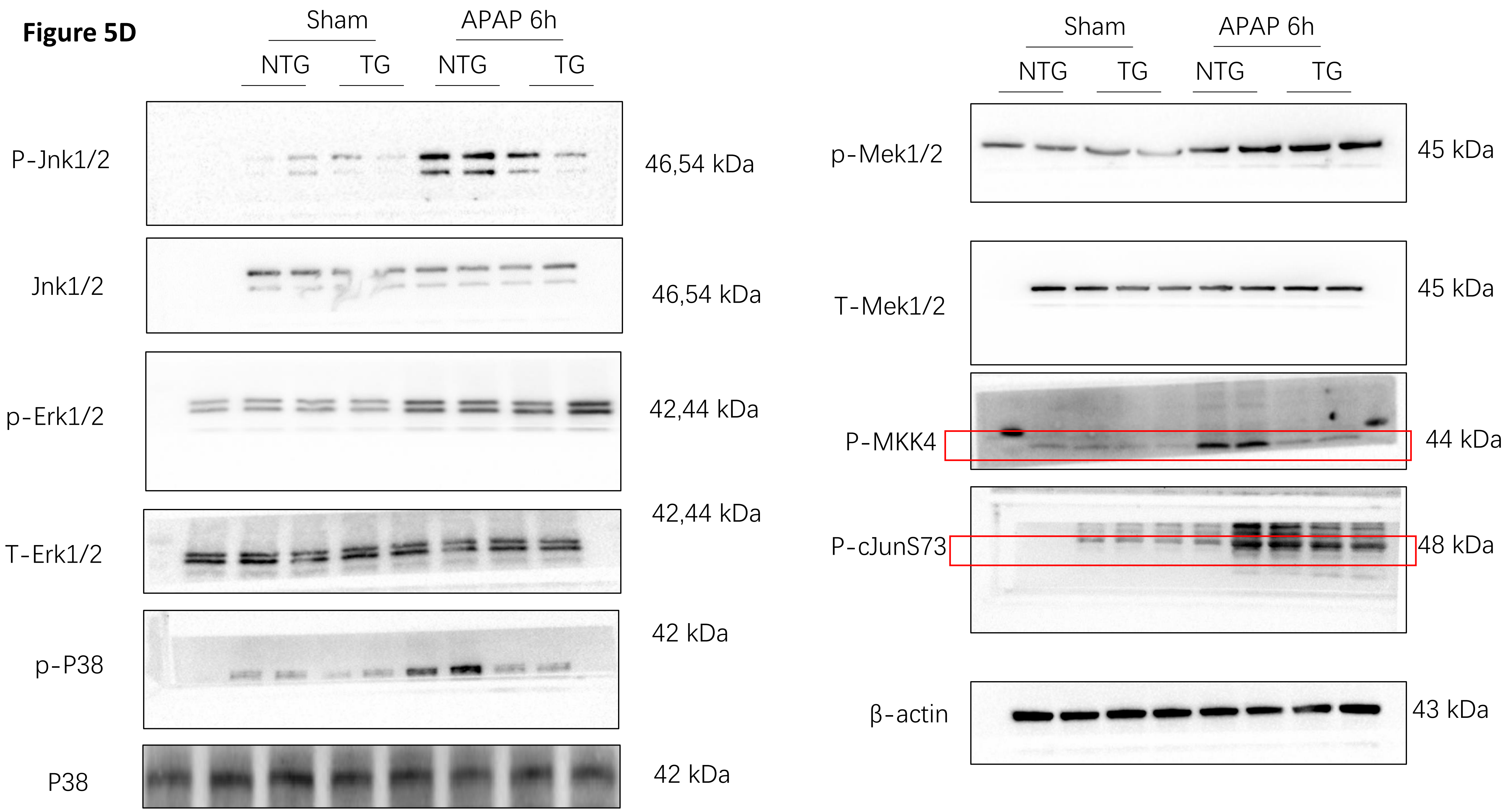
Sham
APAP 6h
NTG
TG
NTG
TG
P-Jnk1/2
46,54 kDa
Jnk1/2
46,54 kDa
p-Erk1/2
42,44 kDa
T-Erk1/2
42,44 kDa
p-P38
42 kDa
P38
42 kDa
Sham
APAP 6h
NTG
TG
NTG
TG
p-Mek1/2
45 kDa
T-Mek1/2
45 kDa
P-MKK4
44 kDa
P-cJunS73
48 kDa
β-actin
43 kDa

Figure 6B

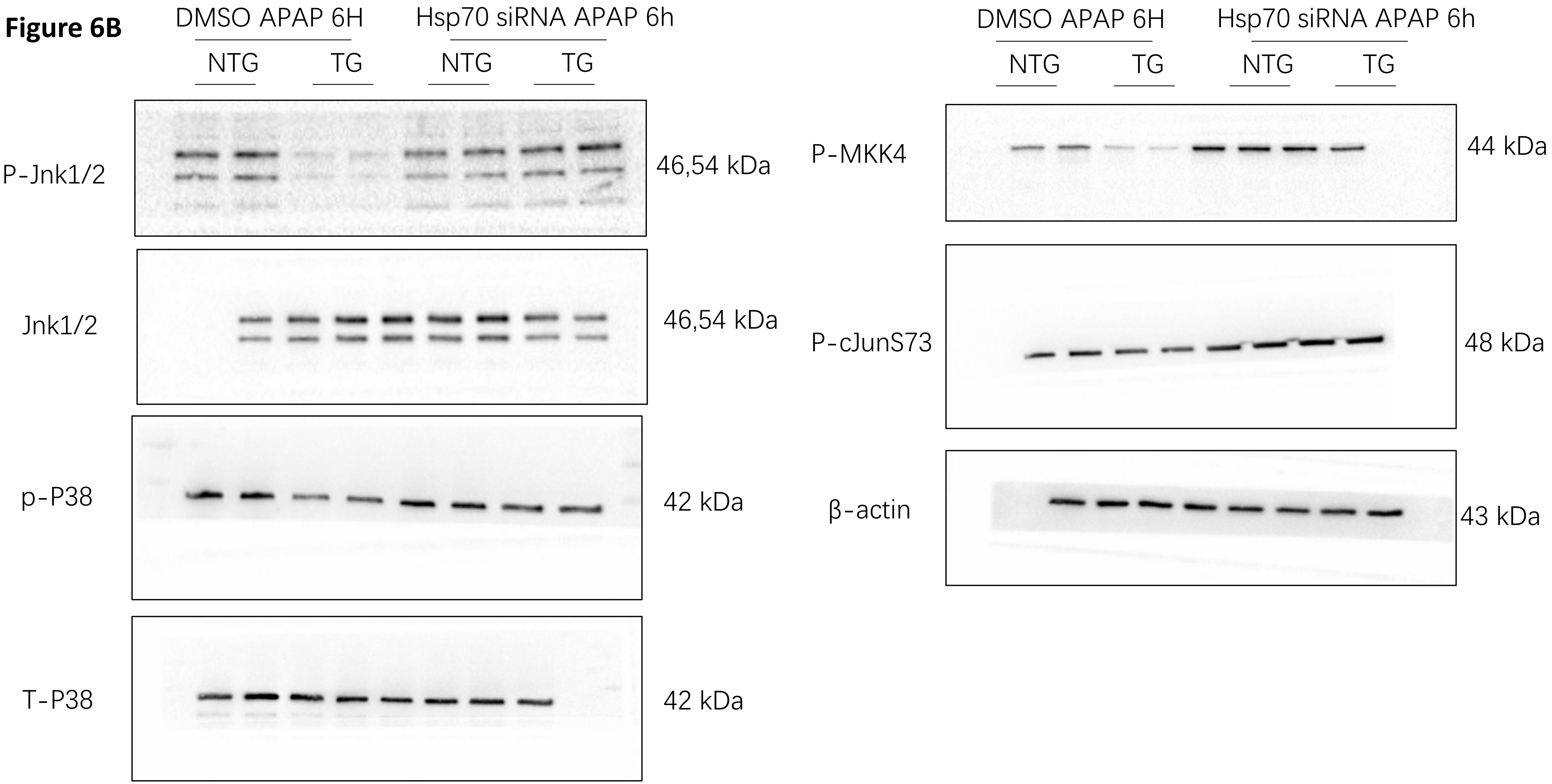
DMSO APAP 6H
Hsp70 siRNA APAP 6h
NTG
TG
NTG
TG
P-Jnk1/2
46,54 kDa
Jnk1/2
46,54 kDa
p-P38
42 kDa
T-P38
42 kDa
DMSO APAP 6H
Hsp70 siRNA APAP 6h
NTG
TG
NTG
TG
P-MKK4
44 kDa
P-cJunS73
48 kDa
β-actin
43 kDa
